# 4-nitroquinoline 1-oxide-induced oral epithelial lesions exhibit time- and stage-dependent changes in the tumor immune microenvironment

**DOI:** 10.3389/fonc.2024.1343839

**Published:** 2024-05-15

**Authors:** Kjersti Sellæg, Ruth Schwienbacher, Mathias Kranz, Anna Engan Aamodt, Anna M. Wirsing, Gerd Berge, Elin Hadler-Olsen, Synnøve Norvoll Magnussen

**Affiliations:** ^1^ Department of Medical Biology, Faculty of Health Sciences, University of Tromsø (UiT) – The Arctic University of Norway, Tromsø, Norway; ^2^ Department of Clinical Pathology, University Hospital of North Norway, Tromsø, Norway; ^3^ PET Imaging Center Tromsø, University Hospital of North Norway, Tromsø, Norway; ^4^ The Public Dental Health Competence Center of Northern Norway, Tromsø, Norway

**Keywords:** 4-nitroquinoline 1-oxide (4NQO), tumor infiltrating lymphocytes (TILs), high endothelial venules (HEVs), tumor microenvironment, tertiary lymphoid structures (TLS), oral carcinogenesis

## Abstract

Oral tongue squamous cell carcinoma (OTSCC) is the most common cancer of the oral cavity and is associated with high morbidity due to local invasion and lymph node metastasis. Tumor infiltrating lymphocytes (TILs) are associated with good prognosis in oral cancer patients and dictate response to treatment. Ectopic sites for immune activation in tumors, known as tertiary lymphoid structures (TLS), and tumor-associated high-endothelial venules (TA-HEVs), which are specialized lymphocyte recruiting vessels, are associated with a favorable prognosis in OSCC. Why only some tumors support the development of TLS and HEVs is poorly understood. In the current study we explored the infiltration of lymphocyte subsets and the development of TLS and HEVs in oral epithelial lesions using the 4-nitroquinoline 1-oxide (4NQO)-induced mouse model of oral carcinogenesis. We found that the immune response to 4NQO-induced oral epithelial lesions was dominated by T cell subsets. The number of T cells (CD4+, FoxP3+, and CD8+), B cells (B220+) and PNAd+ HEVs increased from the earliest to the latest endpoints. All the immune markers increased with the severity of the dysplasia, while the number of HEVs and B cells further increased in SCCs. HEVs were present already in early-stage lesions, while TLS did not develop at any timepoint. This suggests that the 4NQO model is applicable to study the dynamics of the tumor immune microenvironment at early phases of oral cancer development, including the regulation of TA-HEVs in OTSCC.

## 1 Introduction

Oral tongue squamous cell carcinoma (OTSCC) is the most common oral malignancy. OTSCCs are aggressive due to local invasion and a tendency for early metastatic spread to regional lymph nodes (1). About half (47-52%) of the patients will die within five years after diagnosis (2, 3). The standard treatment includes surgery, and in some cases radiotherapy and chemotherapy (2).

A high density of tumor infiltrating lymphocytes (TILs) is associated with good prognosis in several cancers including melanoma, breast, colorectal, and tongue cancers (4, 5). TILs can be separated into B cells and T cells, which can be further classified into various subtypes with specific functions. CD8+ cytotoxic T cells can kill malignant cells and their presence in tumors is associated with favorable patient outcomes (6, 7). These cells are also key players in cancer immunotherapy. Indeed, boosting the presence and reactivity of cytotoxic T cells within tumors, either by blocking of immune checkpoint molecules or transplanting engineered tumor-specific T cells have shown improved clinical outcomes in human cancers (8, 9). Tumor infiltrating B cells have also been shown to correlate with improved patient survival (5), whereas regulatory T cells (Tregs), an immunosuppressive T cell subset, confers worse prognosis in oral cancer patients (10).

In inflamed tissues, B cells, T cells and specialized stromal cells (follicular dendritic cells; FDCs) sometimes form organized immune aggregates known as tertiary lymphoid structures (TLS). The structure and function of TLS resemble lymphoid follicles and act as ectopic sites for local antigen presentation and immune activation (11). Lymphocyte trafficking into lymph nodes and TLS is mediated by specialized post-capillary venules termed high-endothelial venules (HEVs) (12). HEVs with similar phenotypes to lymph node HEVs are found in chronically inflamed tissues, such as autoimmune diseases (13), allograft rejection (14), and solid tumors (15), and are considered the main gateways for lymphocyte entry into these sites. The presence of TLS and HEVs, as well as high density of HEVs within TLS, are associated with a favorable prognosis in several cancers (16–18). In our previous studies we have found that tumor-associated (TA) TLS were associated with a favorable prognosis in OSCC patients (19). Interestingly, TA-HEVs were found to be an independent positive prognostic marker associated a favorable immune microenvironment and were sometimes present independently of TLS (19–21). The specific conditions within a tumor that supports the development of TLS and HEVs remain unclear.

To study OTSCC carcinogenesis and its immune infiltrate, we chose the 4-nitroquinoline 1-oxide (4NQO) mouse model which has been widely used in studies of oral cancer (22). The genetic alterations caused by 4NQO exposure resemble that of tobacco carcinogens (23), which is an important risk factor for oral cancer in humans. To increase our understanding of the immune response in human cancer we need models that closely mimic clinical features of human disease. Our previous studies on TLS and HEVs in OSCC used human archival tissues (19–21). However, archival tissues are snapshots and do not capture the development of the microenvironment during tumor progression. In this study we aimed to determine whether HEVs and TLSs develop in the 4NQO model and to map the immune infiltrate during oral carcinogenesis. We also wanted to explore whether positron emission tomography (PET) and magnetic resonance imaging (MRI) are suitable to track the development of tongue lesions and metastasis in this model. We used immunohistochemistry together with a refined scoring approach to analyze the quantity and spatial distribution of CD4+ (T helper cells), FoxP3+ (regulatory T cells), CD8+ (cytotoxic T cells), and B220+ (B cells) cells, as well as PNAd+ HEVs in tongue tissues. We also performed *in vivo* whole-body PET/MRI of 4NQO-exposed mice at different timepoints. Aggregates of B220+ cells indicative of TLS did not develop at any timepoint, however, HEVs were present already in early-stage lesions. The number of infiltrating lymphocytes and HEVs associated with oral epithelial lesions increased with time and severity of the lesions. PET/MRI efficiently detected epithelial lesions of the tongue and reactive changes in the regional lymph nodes of 4NQO-exposed mice.

## 2 Materials and methods

### 2.1 Animals and experimental protocol

**Figure 1:**
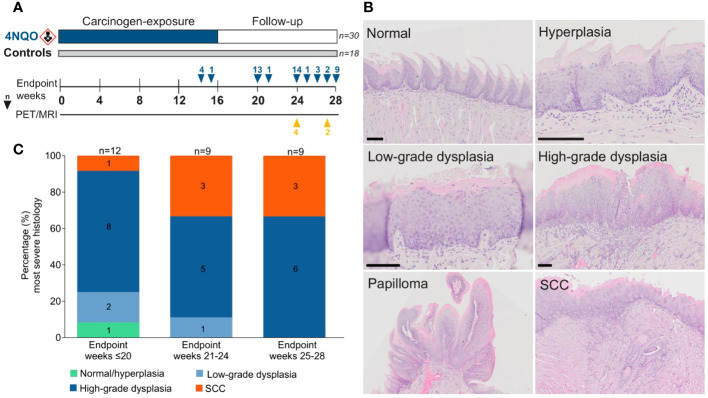
Histopathological evaluation in the 4NQO mouse model. **(A)** Forty-eight mice were given 4NQO-water (n=30) or regular drinking water (n=18) for 16 weeks, followed by 12 weeks observation where the mice were sacrificed at different timepoints. **(B)** Each tongue was assigned a score corresponding to the lesion with the most severe histopathological grade: normal/hyperplasia, low-grade dysplasia, high-grade dysplasia, or SCC. Scale bar indicates 100 µm. **(C)** The severity of epithelial lesions increased in mice sacrificed at later endpoints.

Animal experiments were approved by The Norwegian Food Safety Authority (FOTS ID 15956) and adhere to the Norwegian Animal Welfare Act and the European Union directive 2010/63. For the duration of the study, the mice were maintained at controlled temperature and humidity under a 12h/12h light/dark cycle with *ad libitum* food and water access. The mice were housed at specific-pathogen free conditions and environmental enrichment was provided. Forty-eight C57BL/6 wild-type (strain:C57BL/6JRj) mice (female, 6-8 weeks; Janvier Labs, Route de Genest, France) were kept in ventilated cages (maximum five mice in each cage) with or without filtertops depending on carcinogen exposure. The mice were acclimatized for one week prior to study start. During a treatment period of 16 weeks, mice in randomly selected cages were given either 100µg/mL of the carcinogen 4-nitroquinoline 1-oxide (4NQO, Sigma, St. Louis, Missouri, USA) in the drinking water (4NQO n=30) or regular drinking water (control n=18) (**Figure 1A**). Freshly prepared 4NQO-water was provided every five ± three days. The 4NQO stock solution and drinking bottles were protected from light. During a 12-week follow-up period, carcinogen-exposed and control mice were sacrificed at different timepoints (weeks ≤20: 4NQO n=12, control n=6, weeks 21-24: 4NQO n=9, control n=6, and weeks 25-28: 4NQO n=9, control n=6), referred to as experimental endpoint (**Figure 1A**). Humane endpoints were ≥10% loss of bodyweight and signs of decreased well-being of the animals, including physical appearance and behavior, if it could not be alleviated with intervention. The mice were weighed at baseline (week 0) and weekly until week 14, whereafter the animals were weighed daily until they reached humane or experimental endpoint. Due to loss of body weight and dehydration, the carcinogen-exposed mice were given chow soaked in water in addition to dietary supplements (DietGel^®^ Recovery, Clear H_2_O, Maine, USA) from week 14. Whole-body dynamic PET and high-resolution head MRI was conducted at weeks 24 and 27 as described in detail below. Euthanasia was conducted by carbon dioxide inhalation. Investigators were not blinded to the allocation of animals in the control and experimental group.

### 2.2 Tissue preparation

Immediately after euthanasia, the tongue, cervical and inguinal lymph nodes, lungs, liver, kidneys, and urinary bladder, were collected and fixed in 4% buffered formalin overnight. Thereafter the tissue was kept in 70% ethanol until dehydration and paraffin embedding at the Department of Clinical Pathology, University of North Norway, Tromsø. Before embedding in paraffin, tongues were cut through the midline (medial sulcus) and mounted to give sagittal sections (displaying the tongue from tip to base). Four-µm-thick serial sections were cut from each paraffin-embedded tissue block and mounted on Superfrost Plus glass slides. Sections were coated with paraffin and stored at 4°C until use.

### 2.3 Histopathological evaluation

For histopathological examination of the tongue, one sagittal section from the medial part of the tongue and one from a more lateral part (approximately 80µm apart) were stained with hematoxylin and eosin (H&E). The sagittal sections were used to assess the histology of the whole tongue. Cervical lymph nodes were serial sectioned, and sections at 40µm intervals were H&E-stained to assess local metastatic disease and to verify PET/MRI findings. Furthermore, H&E-stained sections from inguinal lymph nodes, lungs, liver, kidneys, and urinary bladder (one from each organ) were assessed for tumors (distant metastases or primary tumors). H&E staining was done according to standard procedures. Briefly, tissue sections were dewaxed by incubation at 60°C for at least three hours before immersion in xylene, followed by rehydration in graded ethanol baths. The sections were then stained by immersion in Harris hematoxylin (RAL diagnostics, Martillac, France), Scott’s solution (in-house), and eosin (Sigma-Aldrich, St. Louis, Missouri, USA) for 30, 15, and 30 seconds, respectively. An Olympus VS120 automated slide scanner (Olympus, Tokyo, Japan) was used for scanning of stained slides at 20x magnification. Digital virtual whole-slide images were assessed in OlyVIA Image Viewer software (version 2.6) via the Olympus Net Image Server SQL at 0.31- 40x magnification. In case of poor focus of digital slides, the glass slides were studied in a Leica DM2000 light microscope (Leica Microsystems, Wetzlar, Germany).

To map the spatial histology of the tongue we applied an approach adapted from Vered and colleagues (24), where the tongues were divided into seven sectors by imaginary lines; one horizontal line separating the tongue into a dorsal half and a ventral half from tip to base, crossed by three vertical lines. The vertical lines separated the tongue into the following regions: sector 1; dorsal tip, sector 2; ventral tip, sector 3; dorsal anterior body, sector 4; ventral anterior body, sector 5; dorsal posterior body, sector 6; ventral posterior body, sector 7; tongue base. The histology of each tongue was evaluated and assigned to the specific sector. The tongue tissues were scored as normal mucosa, hyperplasia, low-grade dysplasia, high-grade dysplasia, and SCC (**Figure 1B**) according to the WHO classification of head and neck tumors 4^th^ edition (25). Squamous hyperplasia was defined as the presence of a thickened epithelium in the absence of histological criteria for dysplasia (26). Because squamous hyperplasia was observed in both study groups it was considered a normal finding, and therefore, normal mucosa and hyperplasia was grouped together. Epithelial dysplasia was classified as either low- or high-grade using a simplified binary system (26). In low-grade dysplasia, the dysplastic features were restricted to the lower third of the epithelium, and in high-grade dysplasia the dysplastic features were present in the middle- to upper-third of the epithelium. SCC was defined as neoplastic squamous epithelium that penetrated the basement membrane and infiltrated the underlying submucosa. Exophytic lesions composed of acanthotic squamous epithelium arranged along a fibrovascular stem were referred to as papilloma. Histological evaluations of all study animals were performed by KS and clinical pathologist (head- and neck specialist) RS. First, a histopathological examination of the medial tongue sections was performed, during which RS was blinded to the study groups. Next, we repeated the process for the lateral tongue sections. Finally, each tongue and each of their seven sectors were assigned a score corresponding to the most severe epithelial lesion found in the two tissue sections.

### 2.4 Immunohistochemical staining

Consecutive serial sections (within the distance between the two sagittal H&E-stained sections used for histological assessment) were stained for immune cell markers (CD4, FoxP3, CD8, and B220), the HEV specific marker PNAd and the proliferation marker Ki-67. Cervical lymph node sections from mice that underwent PET/MRI were also stained for Ki-67. Specifications for immunohistochemical staining are listed in **Table 1**. Dewaxed and rehydrated tissue sections were subjected to heat-induced antigen retrieval for 20 minutes. After blocking of endogenous peroxidase activity, 5% goat serum was added to prevent non-specific binding of primary antibody. Sections were incubated with primary antibody before adding secondary antibody. Visualization was done with DAKO EnVision DAB+ kit (Aglient, Santa Clara, CA) and counterstain with Hematoxylin (RAL Diagnostics, Martillac, France). Mouse spleen and lymph node tissue was used as positive staining control and primary antibody was omitted for the negative control.

**Table 1:** Specification for immunohistochemical staining.

Target/Primary antibody/Clone	Antigen retrieval	Blocking	Dilution	Incubation time (minutes)	Secondary antibody
T helper cells/Recombinant rabbit monoclonal anti-CD4 antibody/EPR19514^a^	Tris-EDTA (pH 9.0)	0.3% H_2_O_2_	1:1000	60	HRP-labelled polymer anti-rabbit^f^
Cytotoxic T cells/Recombinant monoclonal rabbit anti-CD8 alpha antibody/EPR21769^a^	Sodium citrate (pH 6.0)	0.3% H_2_O_2_	1:2000	ON	HRP-labelled polymer anti-rabbit
T regulatory cells/Rabbit anti-mouse FoxP3 antibody/D6O8R^b^	Sodium citrate (pH 6.0)	3% H_2_O_2_	1:75	ON	HRP-labelled polymer anti-rabbit
B cells/Rat anti-mouse B220/CD45R antibody/RA3-6B2^c^	Tris-EDTA (pH 9.0)	0.3% H_2_O_2_	1:600	60	Goat anti-rat HRP-conjugated (1:200)^g^
High-endothelial venules/Purified Rat anti-mouse/human PNAd antibody/MECA-79^d^	Sodium citrate (pH 6.0)	0.3% H_2_O_2_	1:25	30	Goat anti-rat HRP-conjugated (1:200)
Ki-67/Ki-67 recombinant rabbit monoclonal antibody/SR00-02^e^	Sodium citrate (pH 6.0)	3% H_2_O_2_	1:2000	60	HRP-labelled polymer anti-rabbit

a Abcam, Cambridge, UK;

b Cell Signaling Technology, Dovers, Massachusetts, US;

c R&D Systems, Minneapolis, Minnesota, US;

d BioLegend, San Diego, California, US;

e Invitrogen, Waltham, Massachusetts, US;

f Agilent, Santa Clara, CA;

g Merck, Rahway, New Jersey.

### 2.5 Immunohistochemical evaluation

Immunohistochemically stained sections were assessed using the OlyVIA Image Viewer Software as described for histopathological evaluation. Each of the tongue specimens were divided into sectors (as described above) using the corresponding H&E-stained section as a guide to ensure the sectors matched as accurately as possible. We counted the total number of the various immune cell types in the epithelium and lamina propria of normal mucosa, as well as in and around epithelial lesions of the seven sectors. To evaluate the presence of TLS, we based our scoring approach on methods previously used in human OSCC tumors (19, 27). B220-stained sections were assessed for the presence of distinct aggregates of positively stained cells or more diffuse B220 staining patterns. The presence of CD4+ and CD8+ T cells, as well as PNAd-positive vessels within or adjacent to B220+ cell aggregates on consecutive sections would be considered TLS. Due to the limited presence of HEVs compared to immune cells in the tongue specimens, HEVs were analyzed in two sections per mouse (one medial and one more lateral section, approximately 80µm apart). To report the results, we chose the section that had the highest HEV count out of the two (**Supplementary Figure S1**). HEVs were defined as PNAd-stained vessels or clusters of >1 positively stained cells as previously described (Wirsing 2016). Qualitative assessment of Ki-67 staining was performed in 25 tongue sections (4NQO n=17, control n=8). This included the four carcinogen-exposed- and two control mice that underwent PET/MRI. For these mice, Ki-67 was also evaluated in three cervical lymph node sections at 120µm intervals. Immune cells and Ki-67 staining were analyzed by KS, and HEVs were analyzed by KS and AMW.

### 2.6 Imaging and monitoring with PET/MRI

Animals (4NQO n=4, control n=2) were anesthetized by inhaling 4% Isoflurane (in O_2_), after which anesthesia was maintained at 1.8-2% for up to two hours during imaging procedure. The animals were placed prone on a dedicated heated mouse holder (MINERVE, Esternay, France) and 60 min whole-body dynamic PET imaging (PET/MRI 7T, MR solutions, Guildford, UK) was performed following i.v. application of 6.3 ± 0.3 MBq [^18^F]-fluorodeoxyglucose ([18F]FDG). Simultaneously, high resolution head MRI was performed in the same device applying T1- and T2-weighted fast spin echo (FSE). The radiosynthesis of [18F]FDG was performed according to standard clinical procedure at the PET Imaging Center Tromsø and approximately 1 GBq of the patient batch was used for the animal studies.

The list-mode data were reconstructed into 24x5 s -8x60 s, 10 and x300 s time frames using 3D ordered subset expectation maximization with 1 iteration, 32 subsets, VOXEL size 0.42 mm, applying correction for random coincidences-, decay-, deadtime- and scatter-correction. Subsequently, the hyper-intense lesion was segmented (PMOD v4.3, PMOD Technologies, Zurich, Switzerland) by placing a region of interest (ROI) using the MR information.

### 2.7 PET data analysis

The MRI-based ROI was used to extract the [18F]FDG activity concentration (Bq/ml) on the PET data. PET pharmacokinetic modeling was applied using the irreversible 2TCM (PMOD v.4.3, PMOD technologies) and its suitability evaluated by Schwartz Criterion (SC), Akaike Information Criterion (AIK) and Model Selection Criterion (MSC). Furthermore, rate constants were calculated for K1, k2, k3, and macro parameters MRGlu and Flux derived. The image derived input function (idIF) was segmented from the inferior vena cava as it provides a robust estimation of the idIF (28) with special regard to the spill-in contamination from neighboring tissue when using the heart (29).

### 2.8 Statistical analysis

GraphPad Prism version 10.0.2 (https://www.graphpad.com/) was used for statistical analyses and graphical visualization of data. Quantile-quantile (QQ) plots and the Shapiro-Wilk test were used to assess sample distributions. Mann-Whitney U test or Kruskal-Wallis H test (with Dunn’s multiple comparisons test) was used to analyze difference between two or more groups, respectively, of non-normal distributed data. Student’s t-test or one-way ANOVA (with Tukey’s *post hoc* test for multiple comparisons) was applied to analyze difference between two or more groups, respectively, when the data met the assumption of normal distribution. Statistical analyses were performed using two-tailed tests. Data were transformed for visualization purposes in some instances by log2(y+1). P-value <0.05 was considered statistically significant. In all graphs, asterisks indicate significant differences: *p<0.05, **p<0.01, ***p>0.001, and ****p<0.0001.

## 3 Results

### 3.1 Histopathological characterization of the 4NQO mouse model of oral carcinogenesis

In the current study we used the 4NQO mouse model to analyze the time- and stage-dependent development of the immune infiltrate during oral carcinogenesis. A total of 30 4NQO-exposed mice and 18 healthy controls were sacrificed at different timepoints during the study and were grouped by the weeks at which they reached humane- or experimental endpoint; weeks ≤20 (4NQO: n=12, controls: n=6), weeks 21-24 (4NQO: n=9, controls: n=6), and weeks 25-28 (4NQO: n=9, controls: n=6; **Figure 1A**). The tongue mucosa of most control animals displayed normal histology, while a few mice exhibited features of squamous hyperplasia. Therefore, normal- and hyperplastic epithelium were categorized as one group. 4NQO exposure often induced multiple lesions of varying severity on the same tongue, including low- and high-grade dysplasias, SCC, and papilloma (**Figure 1B**), and each tongue, as a whole, was assigned a score based on the most severe histopathology observed. The severity of the histopathological lesions clearly increased with later endpoints (**Figure 1C**). Only one (3.3%) of the carcinogen-exposed mice had no histopathological lesions, and this animal was sacrificed at the earliest endpoint. High-grade dysplasias were the most common lesions across all endpoints, accounting for 63.3% (19/30) of all cases. At endpoint weeks 25-28, all 4NQO-exposed tongues had either high-grade dysplasia or SCC. Seven 4NQO-exposed mice (23.3%) developed SCC; one at week 20, three at weeks 21-24, and three at weeks 25-28. Half (15/30) of the mice developed one or more papilloma, presenting with either dysplasia or SCC (**Supplementary Figure S2**; **Supplementary Table S1**). In two animals we histologically verified the presence of lesions at other sites in the oral cavity: one in the hard palate and one in the throat. Because the tissues contained only parts of the lesions and surrounding tissue, invasive growth and stage could not be determined. In conclusion, the severity of epithelial lesions increased with time after 4NQO-exposure.

### 3.2 The immune infiltrate gradually increased during oral carcinogenesis and the composition differed with stage

**Figure 2:**
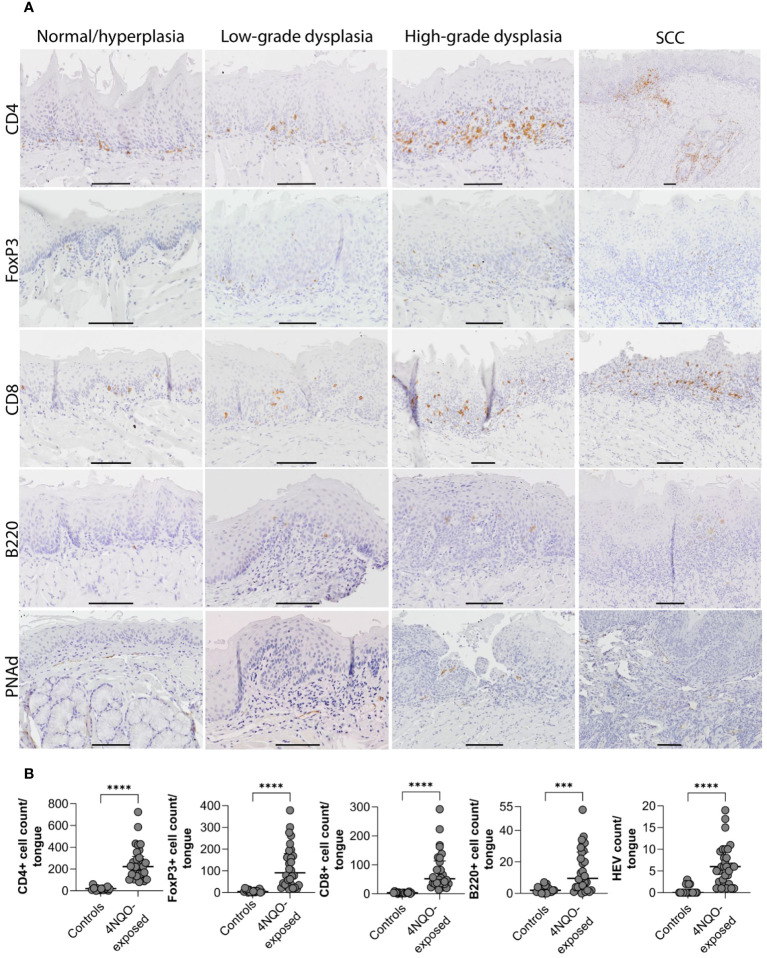
Distribution of immune cells and HEVs in relation to epithelial lesions on the tongue of 4NQO-exposed mice. **(A)** Four µm thick sagittal tongue sections (scale bar indicates 400µm) were stained with H&E and divided into seven sectors (1–7) to **(B)** map the location of epithelial lesions of the tongue following 4NQO-exposure. Data is presented as percentage, and n represents the number of sectors that were examined across all 4NQO-exposed mice. **(C-G)** Total counts of each of the immune cells and HEVs within the seven sectors of the tongue in 4NQO-exposed mice (n=30) and healthy controls (n=18). Data is log-transformed (log2(y+1)), and error bars indicate median with IQR. **(H)** Total counts of each of the immune cells and HEVs in the seven sectors for all 4NQO-exposed mice. The range in the counts for the respective markers are shown to the right where the white is the lowest count and dark purple is the highest count.

**Figure 3:**
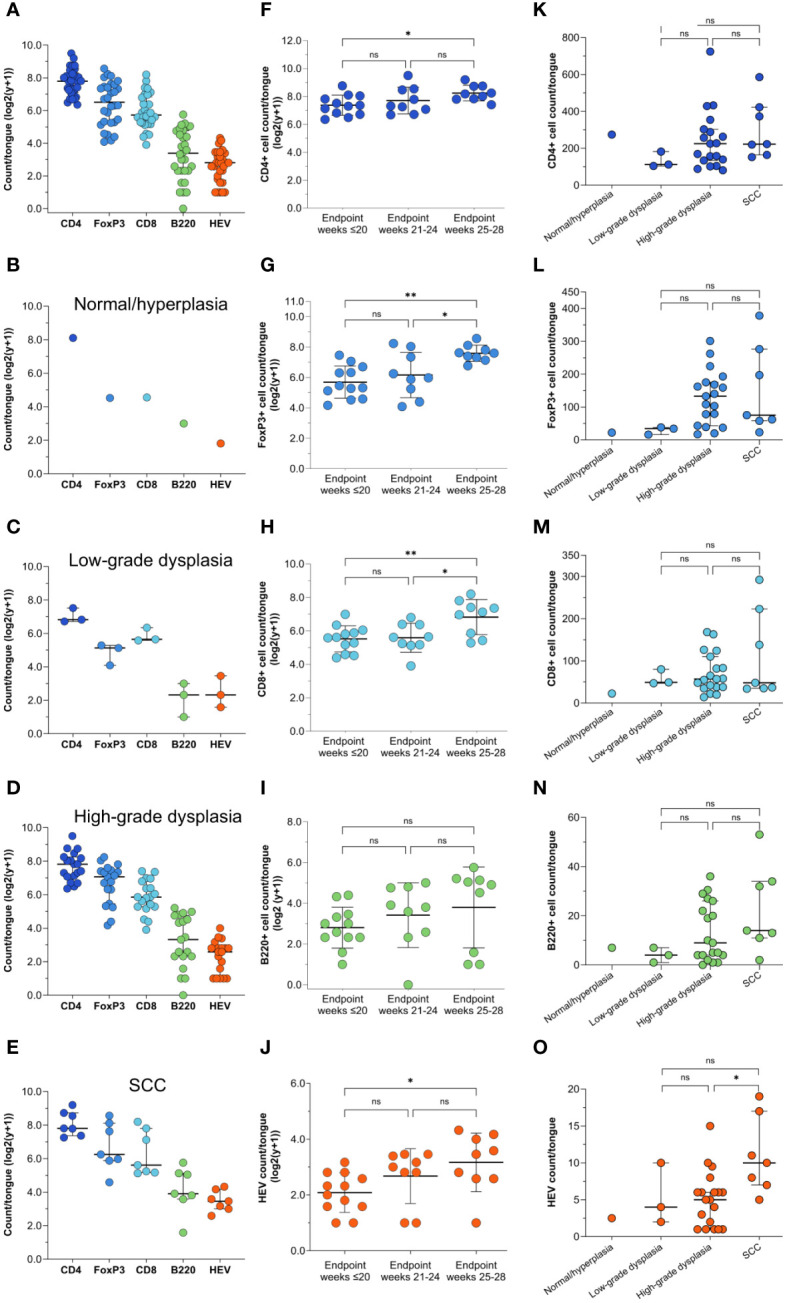
Distribution of immune cell- and HEV markers in the tongue mucosa. **(A)** Shown are representative images for the distribution of CD4-, FoxP3-, CD8-, and B220 cells, and PNAd positive vessels in areas of the tongue graded as normal/hyperplasia, low-grade dysplasia, high-grade dysplasia, and SCC. For CD4, FoxP3, and B220, the same SCC is shown, but the images for FoxP3 and B220 only show parts of the SCC that represented the staining patterns. Scale bar indicates 100µm. **(B)** The number of positively stained cells or vessels for all the markers (CD4, FoxP3, CD8, B220, and PNAd) were significantly higher (P<0.0001) in mice exposed to 4NQO (n=30) compared to healthy controls (n=18). Error bars indicate median with interquartile range (IQR).

To study the immune infiltrate during oral carcinogenesis, whole tongue sections were immunohistochemically stained for the immune cell markers CD4 (T helper cells), FoxP3 (regulatory T cells), CD8 (cytotoxic T cells), and B220 (B cells), and the HEV marker PNAd (**Supplementary Figure S3**). Immune cells were variably found in the epithelium and lamina propria, while HEVs were only found in the stromal compartment typically in areas with several HEVs present, close to epithelial lesions (**Figure 2A**; **Supplementary Figure S4**). For all the markers, the number of positive cells and -vessels were significantly higher in the carcinogen-exposed versus control mice (P ≤ 0.0001) (**Figure 2B**). 4NQO-exposed tongues generally contained sparse numbers of B220+ cells. Aggregates of B220+ cells, which are considered a defining component of TLS (30), were not found in any of the tissue sections analyzed. Irrespective of the severity of the carcinogen-induced epithelial lesions, the most abundant cell type was the CD4+ T cells (**Figures 3A–E**). The total number of positive cells/vessels increased gradually with later endpoints for all the markers (**Figures 3F–J**), though not statistically significant for B220+ cells (**Figure 3I**) (P=0.07). The most prominent increase was seen for FoxP3- and CD8-positive cells (weeks ≤20 n=65 and weeks 25-28 n=205, P=0.0014; weeks ≤20 n=51 and weeks 25-28 n=138, P=0.0071, respectively; **Figures 3G, H**). The CD4+/FoxP3+ cell ratio decreased from the first to the latest endpoint (**Supplementary Figure S5**). Since most FoxP3+ cells also express CD4, this indicates that the proportion of regulatory T cells versus helper T cells increased during tumor progression. The number of HEVs also increased significantly between endpoint weeks ≤20 and endpoint weeks 25-28 (P=0.0292) in carcinogen-exposed mice.

We further assessed if the number of immune cells and HEVs corresponded to the severity of the most severe histopathological lesion (**Figures 3K–O**). Although the differences did not reach statistical significance, the median number of all immune cells increased with the severity of the dysplasia (**Figures 3K–O**). Tongues with SCC had a significantly higher number of HEVs than those with high-grade dysplasia (P=0.0397; **Figures 3K–O**), and the number of B220+ cells increased slightly, although not statistically significant (P=0.2077; **Figure 3N**). CD4+ and CD8+ T cell counts were similar (P=0.3177 and P=0.5984, respectively; **Figures 3K, M**), and the number of FoxP3+ cells was lower (P=0.0741; **Figure 3L**) in tongues with SCC than in tongues with high-grade dysplasias.

### 3.3 A novel scoring approach revealed that the distribution of TILs and HEVs corresponds to the site of the epithelial lesions

**Figure 4:**
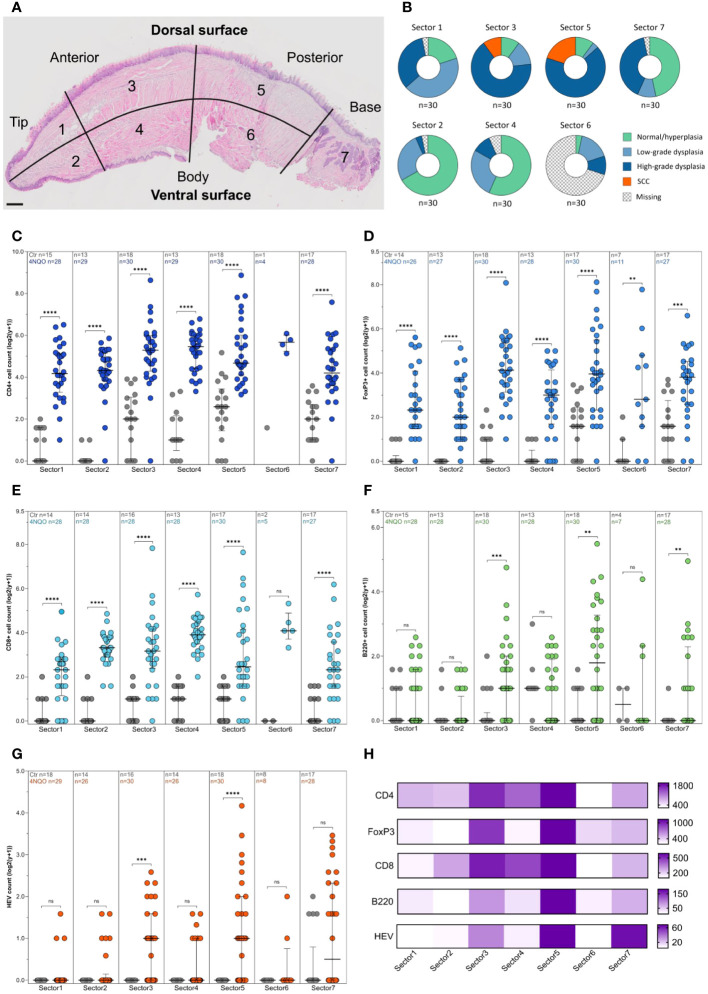
Immune cell and HEV count by endpoint and histopathological grade. **(A)** Shown are the total counts of CD4+, FoxP3+, CD8+ and B220+ cells and HEVs in the tongues of 4NQO-exposed mice (n=30), and the total counts of each of the markers in the tongues graded as **(B)** normal/hyperplasia (n=1), **(C)** low-grade dysplasia (n=3), **(D)** high-grade dysplasia (n=19), and **(E)** SCC (n=7). **(F–J)** The total counts of all the immune cell markers and HEVs in the tongues of 4NQO-exposed mice sacrificed at endpoint weeks ≤20 (n=12), endpoint weeks 21-24 (n=9), and endpoint weeks 25-28 (n=9). **(K–O)** The total counts of immune cells and HEVs in the tongues of 4NQO-exposed mice plotted against the most severe histopathological lesion. Data is log transformed (log2(y-+)). Scale bar indicates median with IQR **(A–E, K–O)** or mean with standard deviation (SD) **(F–J)**.

As previously described, several lesions developed per tongue. Hence, to correlate the immune infiltrate more precisely to the severity of the epithelial lesions, we divided each tongue section into seven sectors and each of them were given a score corresponding to the most severe histopathological change in the sector (**Figures 4A, B**). Altogether, more lesions developed on the dorsal side than on the ventral side of the tongue, and most lesions were found in sectors 3 and 5. These sectors also had the most severe lesions (**Figure 4B**; **Table 2**). SCCs were only found in sector 3 and 5 and accounted for 10% and 20% of the lesions, respectively. The SCCs sometimes extended into both sectors. Large papillomas were most common on the dorsal posterior body of the tongue (sector 5) and tongue base (sector 7) (**Supplementary Figure S2**; **Supplementary Table S1**). The area least affected by 4NQO-exposure was the ventral part of the tongue, in sectors 2 and 4, in which 66.7% and 56.7% of the mice presented with normal/hyperplastic mucosa, respectively. The ventral posterior part of the tongue (sector 6) was missing in many sections because excision of the tongue was performed in this region. The severity of the histopathological lesions increased from the earliest to the last endpoint in all areas of the tongue (**Supplementary Figure S6**).

**Table 2:** Worst histologic grade in sectors 1-7 in the tongue of 4NQO-exposed mice.

	Sector 1	Sector 2	Sector 3	Sector 4	Sector 5	Sector 6	Sector 7
Worst histologic grade	*n=29 (No.(%))*	*n=29 (No.(%))*	*n=30 (No.(%))*	*n=29 (No.(%))*	*n=30 (No.(%))*	*n=9 (No.(%))*	*n=29 (No.(%))*
Normal/hyperplasia	6 (20.0)	20 (66.7)	3 (10.0)	17 (56.7)	3 (10.0)	1 (3.3)	14 (46.7)
Low-grade dysplasia	13 (43.3)	8 (26.7)	4 (13.3)	8 (26.7)	1 (3.3)	5 (16.7)	3 (10.0)
High-grade dysplasia	10 (33.3)	1 (3.3)	20 (66.7)	3 (10.0)	20 (66.7)	3 (10.0)	12 (40.0)
SCC	0 (0.0)	0 (0.0)	4 (10.0)	0 (0.0)	6 (20.0)	0 (0.0)	0 (0.0)
Missing	1 (3.3)	1 (3.3)	0 (0.0)	2 (6.7)	0 (0.0)	21 (70)	1 (3.3)

Lower numbers of immune cells and HEVs were found in all seven sectors of the control tongues compared to 4NQO-exposed tongues (**Figures 4C–G**). Sector 3 and 5 displayed a substantial accumulation of immune cells and HEVs (**Figures 4C–H**), which corresponded with the severity of the epithelial lesions found in these sectors (**Figure 4B**). Interestingly, the median number of CD4+ and CD8+ T cells were highest in sector 4, despite the presence of few lesions in this area (**Figures 4C, E**). While the number of CD4+ T cells in sector 4 was associated with the presence of epithelial lesions (median CD4+ T cells without lesions n=35, and with lesions n=55, P=0.0038), the number of CD8+ T cells was not (P=0.755). For B cells and HEVs, the highest count was found in sector 5 (**Figures 4F, G**). Several tongues also displayed a high HEV count in sector 7. Large papillomas often displayed a strong immune infiltrate, likely reflecting the high numbers of HEVs seen in this area (**Figure 4G**; **Supplementary Figure S2**). Interestingly, sector 7 was the only area of the tongue where HEVs were observed in the control group (**Figure 4G**). This area of the mouse tongue has mucous- and serous salivary glands and we observed that structures characteristic of excretory ducts of the salivary glands were often surrounded by several HEVs and immune cells (**Supplementary Figure S7**), even in the absence of histological changes in 4NQO-exposed mice. We also found HEVs in this area in healthy controls. Additionally, sectors 2 and 4 were the only other sites of the 4NQO-exposed tongues where HEVs were present without any epithelial lesions. Finally, we calculated the number of immune cells and HEVs within sectors assigned the same histopathological score to get a more detailed image of the immune composition closely linked to the epithelial lesions (**Supplementary Figure S8**). In summary, the presence of immune cells and HEVs corresponded with the site and severity of epithelial lesion.

### 3.4 4NQO-exposed mice displayed reactive draining lymph nodes but no metastasis

**Figure 5:**
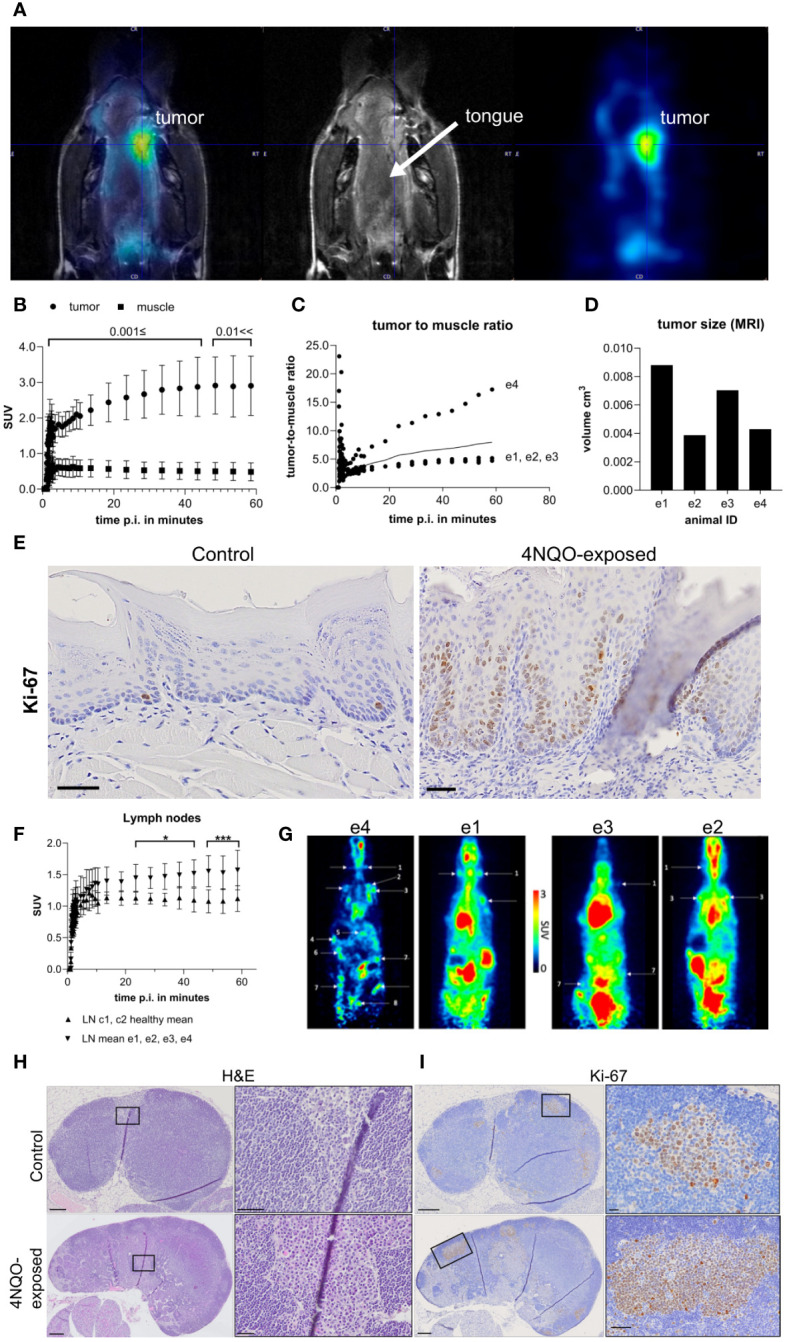
*In vivo* PET/MRI results of mice following i.v. injection of [18F]FDG. **(A)** PET/MRI image of a representative animal (animal e4) representing uptake of [18F]FGD in the hyperintense lesion (left and right). The T2-weightes (middle) shows the lesion on the lateral side of the tongue marked by the crosshair. Overlapping PET and MRI image is shown to the left, while the middle and right shows MRI and PET image, respectively. **(B)** Standardized uptake values (SUV) of the whole lesion (round dots) and leg muscle (square dots, reference tissue), **(C)** lesion-to-muscle ratio in the four carcinogen-exposed mice (e1-e4), and **(D)** volumes of the hyperintense lesions of each animal (e1-e4) derived from T1-weighted MRI. **(E)** Representative images of Ki-67 staining in the (dorsal) tongue of healthy controls and 4NQO-exposed mice. Scale bar indicates 50µm. **(F)** SUV of lymph nodes of 4NQO-exposed (n=4, e1-e4, upward pointing arrowheads) and control mice (n=2, c1-c2, downward pointing arrowheads). Error bars indicate mean with SD. **(G)** PET/MRI results showing SUV of the lymph nodes indicated by arrows/labels in the four 4NQO-exposed mice. Representative images of **(H)** H&E- and **(I)** Ki-67-stained sections of cervical lymph nodes in 4NQO-exposed mice and healthy controls. Detailed images of plasma cells and Ki-67+ cells from outlined areas (black box) are shown in H&E- and Ki-67-stained sections, right panels of **(H, I)**, respectively. Scale bars indicate 200µm **(H, I)** lymph node overview), 50µm **(E, H)** outlined area control and 4NQO, **(I)** outlined area 4NQO) or 20µm **(I)** outlined area control).

A small group of mice (4NQO n=4, control n=2) underwent PET/MRI (**Figure 1A**) to determine whether this combined modality could be used to detect primary- and metastatic lesions as a non-invasive method to follow tumor progression. The mice were tail-vein injected with [18F]FDG, a glucose analogue radiotracer. MRI clearly revealed a hyperintense signal on the tongue, offset to the midsagittal plane, of the four 4NQO-exposed mice and PET confirmed specific [18F]FDG-uptake (**Figure 5A**). The radiotracer accumulation in this area increased over time and had a standardized uptake value of 8.0 ± 6.2 when compared to the muscle reference region, which reached an early plateau (**Figure 5B**). The [18F]FDG-uptake in this region was also distinct from the surrounding tissues, corresponding well with the PET images. The ratio of [18F]FDG-uptake in the hyper-intense lesion vs muscle varied among the different animals, with animal e4 showing the largest deviation from the mean (**Figure 5C**). However, this was not linked to the variation in the size of the lesion, with animal e4 having one of the smallest lesions (**Figure 5D**). This could indicate a difference in metabolic rate and/or cell proliferation.

Following PET pharmacokinetic modeling, the rate constants were expressed as ml/ccm/min (K1, Flux), 1/min (k2-k3) and μmol/min/100g (MRGlu) and the results are shown in **Supplementary Figures S9A, B**. The highest glucose uptake and retention were seen in the hyperintense lesions when comparing to the reference region, confirming increased glucose consumption in these areas of the tongue (**Figures 5A, B**). Histological examination based on one half of the tongue (the other half was used for other purposes) showed that all the four experimental mice had several lesions on the tongue. The most severe lesions in mice e1, e2 and e4 were high-grade dysplasias, while e3 had low-grade dysplastic lesions as the most severe score. Staining for Ki-67 showed no obvious difference in the number of positive cells in the tongue epithelium between these four mice. However, most of the 4NQO-exposed mice (13/17) displayed increased Ki-67 positivity in the tongue compared to healthy controls (n=8), and the distribution of the positively stained cells corresponded with the presence of epithelial lesions (**Figure 5E**).

To assess whether PET could be used to detect lymph node metastasis, an in-depth image analysis of the cervical- and distant lymph nodes was performed. The analysis revealed increased [18F]FDG uptake in the carcinogen-exposed group compared to the healthy controls (**Figures 5F, G**). Animal e4 presented with higher glucose metabolism in all lymph nodes, while e1, e2, and e3 showed active [18F]FDG uptake primarily in the cervical and axillary region (**Figures 5F, G**). To assess whether the observed [18F]FDG uptake was due to lymph node metastasis, H&E-stained serial sections of cervical lymph nodes from the four carcinogen-exposed mice and controls (c1-c2) were assessed for metastatic lesions. The results showed that none of the lymph nodes contained cancer cells (primary or metastatic) (**Figure 5H**). However, when comparing the 4NQO group to the controls, the carcinogen-exposed group had enlarged lymph nodes owing to expansion of the B and T cell compartments. We also observed a prominent accumulation of cells with abundant cytoplasm and an eccentrically located nucleus, indicative of plasma cells in the paracortex and medulla (**Figure 5H**). Animals e1-e3 exhibited low to moderate presence of plasma cells, while in e4, plasma cells comprised nearly half of certain lymph nodes. Ki-67 staining of cervical lymph nodes did show accumulation of positively stained cells in areas corresponding to germinal centers, which were larger in the 4NQO-exposed mice compared to healthy controls (**Figure 5I**). To conclude, no regional lymph node metastases or distant metastases were detected using whole-body scans, nor upon histopathological analysis of the examined tissues (cervical- and inguinal lymph nodes, lungs, liver, kidneys, and urinary bladder).

## 4 Discussion

The current study presents a detailed description of how the tongue epithelial lesions and associated immune infiltrate evolve in the 4NQO mouse model after oral administration of the carcinogen for 16 weeks. We evaluated the potential of PET/MRI as a tool to monitor tumor progression in this model. We showed that 4NQO exposure induced epithelial lesions in the tongue that increased in severity over time, where all mice had high-grade dysplastic lesions or SCCs at the latest timepoint (9 weeks or longer after termination of carcinogen exposure). These findings are consistent with other reports of this model (31–34), and confirm its ability to replicate the multistep carcinogenesis in humans through spontaneous tumor development and progression, alongside a functional immune system. Our results showed that the lymphocytic infiltrate and HEVs increased over time after 4NQO exposure, in concert with the severity of epithelial lesions.

A challenge we faced with this model was the development of multiple lesions with varying severity on the same tongue. Using a mouse model with transplanted cells could provide more predictable and consistent tumor development, however, we were interested in the early development of the tumor and the associated immune response. Genetically engineered models with inducible tissue-targeted expression and/or knockout of oncogenes and tumor suppressor genes could also provide more predictable tumorigenesis but are often challenged by a lack of tissue-specificity and risk of leakage of transgene expression (35). We handled the challenge of multiple lesions by using a scoring method where the tongue was divided into seven sectors. We used a similar approach as Vered et al. (24), in which they separated the tongue of rats into three equal parts: the anterior, middle, and posterior part of the tongue. Because the tongue base in mice contains the lingual salivary glands, which is distinct from the anatomy of the rest of the tongue, we chose to separate this area into a single sector. In support of our approach of grouping lesions from small anatomical regions, Sequeira et al. (32) found that individual 4NQO-induced lesions close to each other were more likely to be clonally related. The clonal origin differed when the lesions were located some distance from each other (32). Thus, our approach can be used to study the stage-by-stage changes that occur in the tongue mucosa.

Neither local nor distant metastasis was observed in any of the 4NQO-exposed mice in our study. This is in line with other studies reporting that lymph node metastasis in this model was infrequent until week 33 post 4NQO exposure (36, 37). Some of the animals had to be sacrificed before reaching the final experimental endpoint due to a high primary tumor burden. Hence, the mice were probably terminated before the development of metastasis. Despite lack of metastases upon histopathological examination, we saw prominent PET signals from the cervical lymph nodes of the 4NQO-exposed mice. These signals were probably due to increased [18F]FDG uptake caused by reactive changes in the lymph nodes indicated by a prominent plasma cell component within the paracortex and medulla, as well as increased proliferation of B cell follicles measured by Ki-67 expression and enlarged T cell zones. Although no metastases were detected in our experiment, the strong PET-signal from reactive lymph nodes suggests that [18F]FDG is an unsuitable tracer for discriminating between metastases and reactive changes in this model. Using PET radiotracers that are more tumor- or metastasis-specific, such as [18F]FAPI-74, [18F]Fluciclovine, or [68Ga]Pentixafor, might enhance the ability to distinguish between reactive changes and metastasis in lymph nodes.

It is well established that the tumor immune infiltrate has a major influence on tumor progression and the response of solid tumors to immunotherapy (4). Similar to our current findings, previous studies on the 4NQO mouse model have demonstrated that changes in the immune infiltrate correspond with the histological grade (38, 39). This is consistent with findings in human tongue specimens of different histological grades (40), as well as across oral subsites (41, 42). Our findings together with other studies indicate that mouse and human oral lesions display a pronounced influx of T cells that increase with more severe histopathology (39, 40, 42). Consistently, OSCCs are often abundantly infiltrated with T cells (43, 44). However, marked B cell infiltrates have been found in human oral cancers (5, 45), which might reflect the presence of TLS. Consequently, tumors that lack TLS tend to display low numbers of B cells (27, 46). In the 4NQO-induced tongue lesions, we found that B cells were present at low numbers. Accordingly, we found low numbers of B cells and no aggregates resembling the formation of TLS in the tongue tissues. Sales de Sà et al. reported that among 48 OTSCC patients, 77.1% were enriched in CD20+ B cells (5). Intratumoral B cells are mostly located in TLS and aggregated with T cells (30). Indeed, the presence of B cells in OTSCC tumors positively correlated with the density of T cells (5), indicative of TLS formation. Although the presence of B cells was low in the 4NQO-exposed tongues, the number of B cells was higher in SCCs compared to earlier stages. This might indicate that TLSs develop at a later timepoint in this model. We have earlier found that TLSs in OSCC are infrequent and heterogeneously distributed (19), and it might be that we have missed TLSs by not examining whole tongues. Alternatively, the presence of a sparse B cell infiltrate could point to a role of B cells in promoting OSCC. There is growing evidence that intratumoral B cells found outside TLS may be involved in tumor progression (47, 48). However, the role of different B cell subsets in OSCC is largely unexplored, and reliable markers for identifying B cell phenotypes remain undefined. Recently, tumor-associated lymphoid aggregates rich in T cells with a stem-cell like phenotype (CD8+TCF1+) and antigen-presenting cells (APCs) has been identified in human tumors and tumor-bearing mice (49, 50). These so-called antigen-presenting niches (APNs) resemble T cell zones in SLOs, suggesting that they may function as intra-tumoral sites for priming and activation of T cells. CD8+TCF1+ T cell niches have also been found in close proximity to TA-HEVs (51). Hence, lymphoid aggregates other than TLS can impact tumor control, however, we have yet to determine the presence of APNs in the 4NQO model. While TILs are often examined in established tumors, the tumor immune response is dynamic and constantly reshaped by interactions with neoplastic cells and the microenvironment at different stages of tumorigenesis (52). Efficient use of animal models warrants a better understanding of the immune infiltrate. Based on these results, the 4NQO mouse model is suitable for studies of T cells responses in oral carcinogenesis and perhaps tumor promoting B cells.

We found that HEVs developed in the tongue already 14 weeks after administration of the carcinogen, even when hyperplasia was the most severe histology. HEVs were found in the tongue of all 4NQO-exposed mice and in some controls. To the best of our knowledge HEVs have not been reported in this model previously. The presence of HEVs has been reported in oral lesions with malignant potential (53, 54), but HEV development at different stages of oral cancer development and progression is not well described. In OSCC, HEVs are more frequently found in early-stage tumors (T1-T2) than advanced tumors (T3-T4) (21, 55). Here we showed that the number of HEVs increased significantly during progression of tongue lesions, suggesting that early changes in the tongue mucosa initiates HEV formation that is maintained during tumor initiation and progression.

In tumors, HEVs are typically found in areas rich in lymphocytes, including TLS. TLSs share some characteristics with lymph nodes, including organized clusters of B and T cells, interspersed with antigen presenting cells and stromal cells (11). HEVs recruit lymphocytes into the TLS, which serve as a local site for adaptive immune responses. The presence of TA-HEVs and TLS is associated with a favorable prognosis in many cancers (15), including OSCC (19, 21, 27). However, the location of TA-HEVs within the tumor microenvironment could further refine their prognostic value. A high HEV density within TLS correlated with longer survival in colorectal cancer patients compared to HEV low TLS (16). These findings demonstrate a close relationship between HEVs and TLS in anti-tumor immunity. Interestingly, the presence of TA-HEVs does not always correlate with the presence of TLS (15, 19, 21). This raises the question whether TA-HEVs within TLS are regulated differently than those independent of TLS, and whether this could impact on their functionality. Compared to homeostatic HEVs in the lymph node, that display a thick vessel wall and small lumen, more heterogenous HEV phenotypes have been found in inflamed lymph nodes and tumors (21, 56). The heterogenous morphology of these HEVs can be explained by differences in gene signatures revealed by transcriptomic analyses (57, 58), in which lymph node HEVs are considered fully differentiated (mature), and the inflamed- and TA-HEVs are dedifferentiated. Despite being dedifferentiated, spontaneously arising TA-HEVs have been shown to harbor some lymphocyte-recruiting capacity (59, 60). In a mouse model of fibrosarcoma, treatment with immune checkpoint inhibitors, (anti-PD-1 and -CTLA-4) induced the frequency and maturation of TA-HEVs and led to increased numbers of infiltrating CD4 and CD8 T cells (60). This suggests some functional impairment of TA-HEVs.

Although it is not well-known what triggers the development of TA-HEVs, some studies have proposed a positive feedback-loop mechanism. In lymph nodes, dendritic cells are recognized as main drivers of HEV formation. However, in murine tumors the presence of an active T cell response prior to or within the developing tumor appears to be essential for TA-HEV development. For instance, B16-OVA tumors grown in Rag^-^/^-^ mice, that lack mature B and T lymphocytes, also lack TA-HEVs (59). Repleting these mice with CD8 T cells induced TA-HEVs. T cell depletion using anti-CD4 and -CD8 antibodies were also shown to reduce the number of TA-HEVs in murine pancreatic neuroendocrine tumors (61). Induction of TA-HEVs by immune checkpoint inhibitors is also a strong indicator of the necessity of reactive T cells (51, 60, 61). Here we show that 4NQO does not induce TLS formation but supports TA-HEV development. Hence, the 4NQO model can be used to study mechanisms regulating HEV neogenesis. The model could furthermore be beneficial for studies aiming to understand mechanisms driving the differentiation and TA-HEVs with the aim of refining anti-cancer therapy.

In conclusion, we have shown that the 4NQO mouse model generates an immune microenvironment that reflects early stages of oral carcinogenesis and constitutes the required signals for HEV formation. While 4NQO-induced lesions display a marked T cell infiltrate, we found sparse numbers of B cells and no B cells aggregates indicating TLS. The present study is the first to report the presence of HEVs within 4NQO-induced oral lesions, making it a promising model to dissect the components involved in generating a permissive milieu for *de novo* HEV development independent of TLS. Whether these vessels have functional capabilities is yet to be answered. Understanding the mechanisms regulating TA-HEVs can permit the development of targeted therapies for oral cancer patients. PET/MRI using [18F]FDG radiotracer is efficient in detecting reactive cervical lymph nodes. Although we did not detect any metastatic lesions in the mice, the strong [18F]FDG signals in reactive lymph nodes suggests that other tracers are needed to detect lymph node metastases. Due to the low incidence of metastasis in this model while the tumor burden is manageable for the mice, the 4NQO model is best used to study early stages of oral carcinogenesis.

## Data availability statement

The raw data supporting the conclusions of this article will be made available by the authors, without undue reservation.

## Ethics statement

The animal study was approved by The Norwegian Food Safety Authority. The study was conducted in accordance with the local legislation and institutional requirements.

## Author contributions

KS: Conceptualization, Investigation, Visualization, Writing – original draft, Writing – review & editing, Data curation, Formal analysis, Methodology, Project administration. RS: Investigation, Writing – review & editing, Formal analysis, Methodology. MK: Investigation, Writing – review & editing, Data curation, Formal analysis, Methodology, Visualization. AA: Investigation, Writing – review & editing, Formal analysis. AW: Methodology, Writing – review & editing, Conceptualization, Project administration, Supervision. GB: Investigation, Writing – review & editing, Conceptualization, Project administration, Supervision. EH-O: Conceptualization, Methodology, Project administration, Supervision, Writing – review & editing, Funding acquisition, Investigation. SM: Formal analysis, Investigation, Methodology, Writing – review & editing, Conceptualization, Data curation, Funding acquisition, Project administration, Supervision, Writing – original draft.
