# Supplementary material for: 4-nitroquinoline 1-oxide-induced oral epithelial lesions exhibit time- and stage-dependent changes in the tumor immune microenvironment
Source: Front Oncol. 2024 May 15;14:1343839. doi: 10.3389/fonc.2024.1343839 (PMC11133644; doi:10.3389/fonc.2024.1343839)
Supplement: Supplementary file 1 [file DataSheet_1.docx]

Supplementary Material

# Supplementary Figures and Tables

## Supplementary Figures


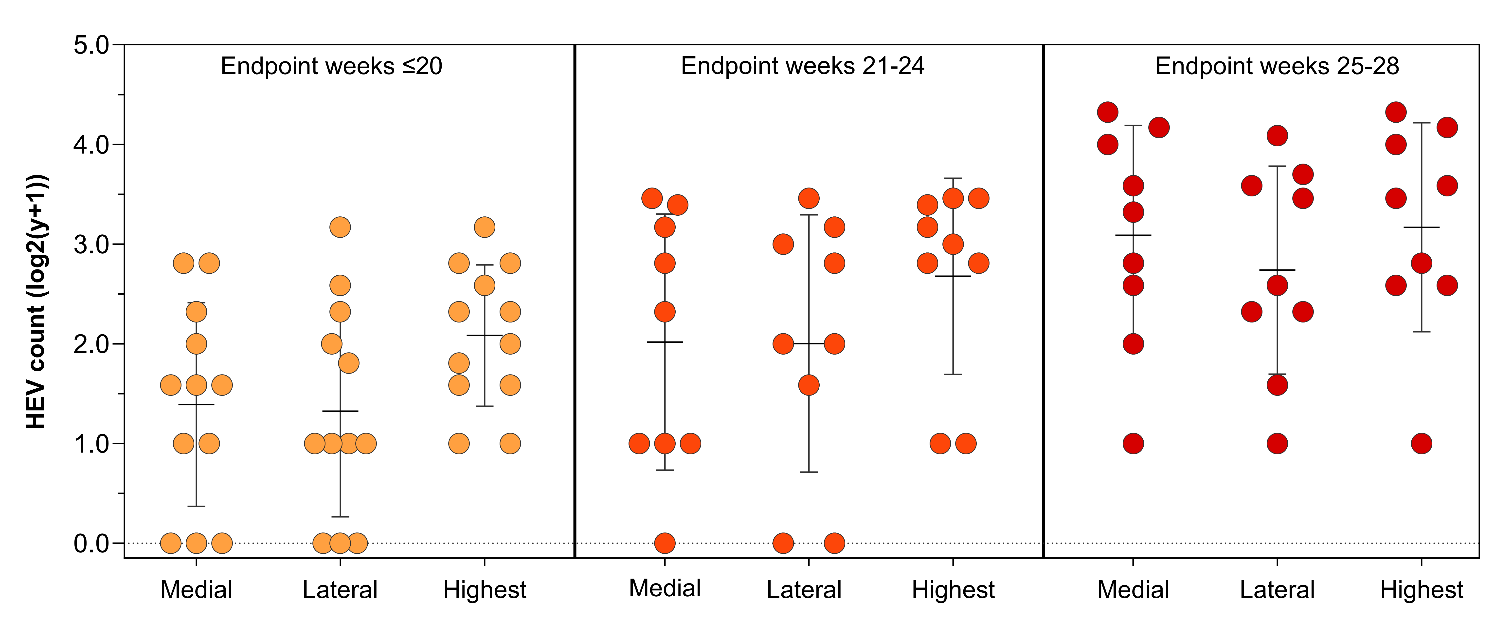


**Supplementary figure S1. Evaluation of HEVs.** The number of PNAd-positive HEVs were evaluated in one medial and one more later section (approximately 80µm apart) for each animal sacrificed at different endpoints (weeks ≤20 n=12, 21-24 n=9, and 25-28 n=9). Due to the limited presence of HEVs in the tongue sections, the section (out of the two; medial and lateral) which contained the highest number of HEVs were selected to present the results. The figure shows the total HEV count in the medial and lateral section of each mouse, and the distribution of the total HEV count in each animal when the section containing the highest number of HEVs was selected. Data is log-transformed (log2(y+1)). Error bars indicate mean with SD.


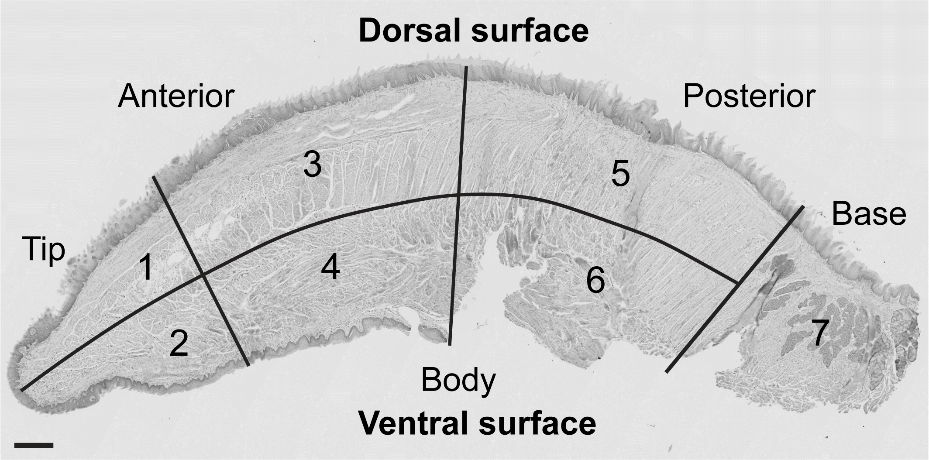

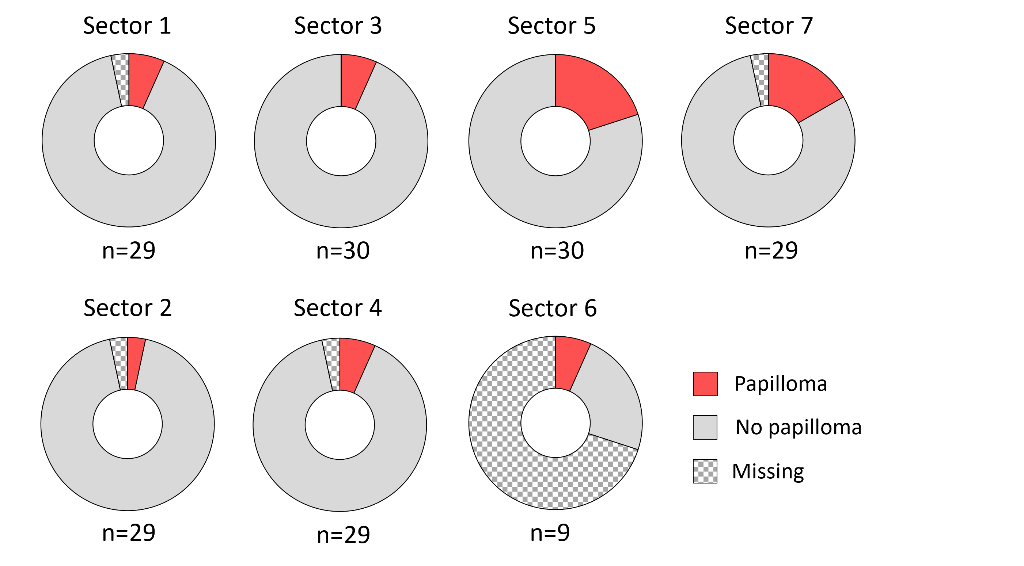


**Supplementary figure S2. Distribution of papilloma in tongue sectors 1-7 in 4NQO-exposed mice.** Tongue tissue sections were separated into seven sectors to assess the location of epithelial lesions. Twenty papillomas presenting with dysplasia or SCC were found in fifteen animals. n represents the number of sectors that were examined across all 4NQO-exposed mice (n=30). Data is presented as percentage (details are listed in table S1). Scale bar indicates 400µm.


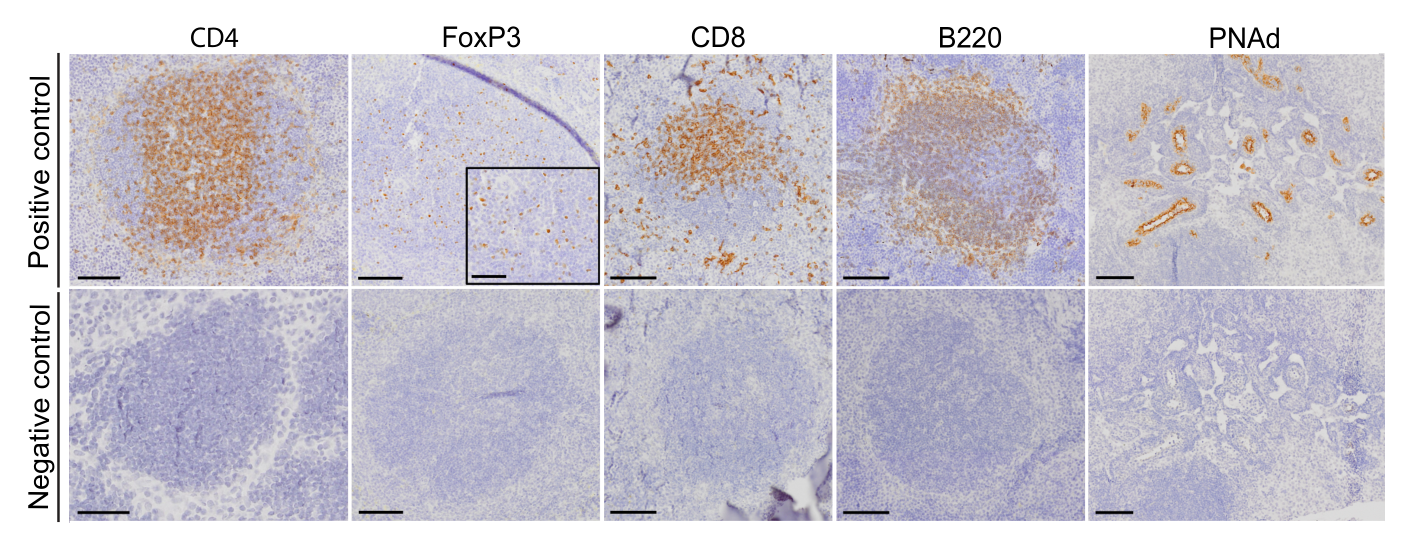


**Supplementary figure S3. Controls for immunohistochemical staining.** Positive and negative controls for immunohistochemical staining of CD4 (CD4 helper T cells), FoxP3 (regulatory T cells), CD8 (cytotoxic T cells), B220 (B-cells), and PNAd (HEVs) were included for each round of staining and were treated identical to the samples. The primary antibody was omitted from the negative control. Mouse spleen was used as control tissue for immune cell markers, while mouse lymph node tissue was used for PNAd. Scale bar indicates 100µm or 50µm (CD4 negative control and FoxP3 outlined box).


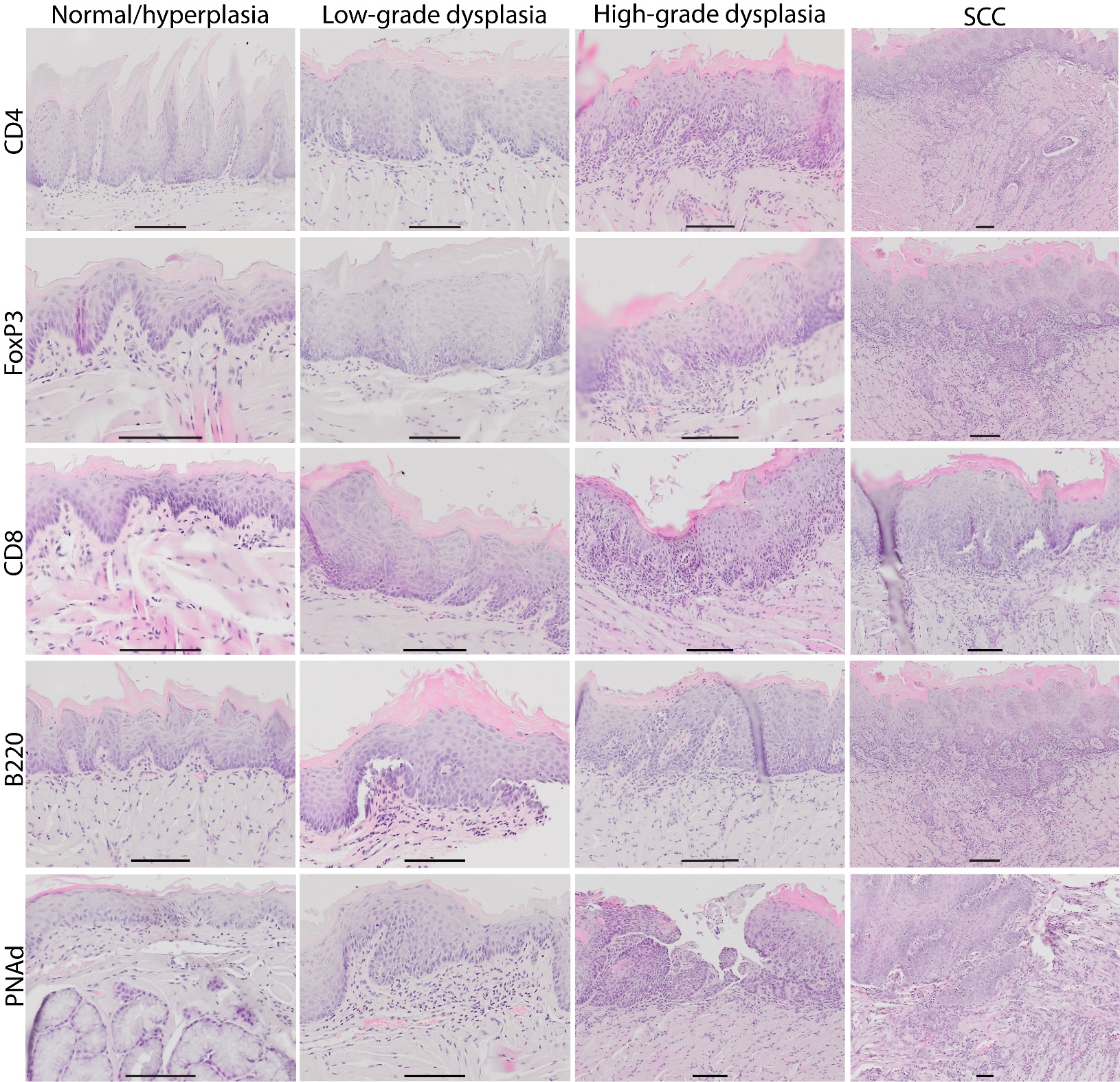


**Supplementary figure S4. H&E-stained sections for histological grading of immunohistochemically stained tissue.** Shown are the corresponding areas in H&E-stained tongue tissue sections to the representative images of each of the markers (CD4, FoxP3, CD8, B220, and PNAd) in figure 2. For CD4, FoxP3, and B220, the same SCC is shown, but the images for FoxP3 and B220 only show parts of the SCC that corresponds to the area shown for the immunohistochemical staining in figure 2. Scale bar indicates 100 µm.


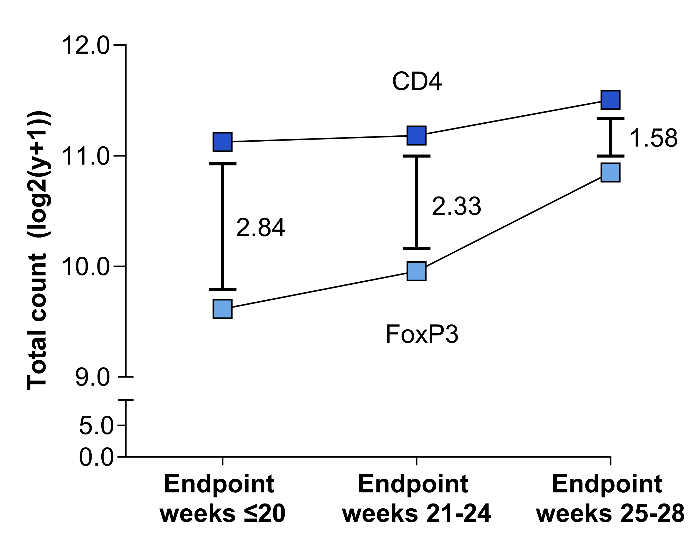


**Supplementary figure S5. Ratio between CD4 T helper cells and regulatory T cells by endpoint weeks.** Shown is the ratio between the total number of CD4+ (dark blue) and FoxP3+ (light blue) cells in 4NQO-mice at endpoint weeks ≤20 (n=12), 21-24 (n=9), and 25-28 (n=9). The ratio is calculated as the total number of CD4+ cells/the total number of FoxP3+ cells at each endpoint. Data is log-transformed (log2(y+1)).


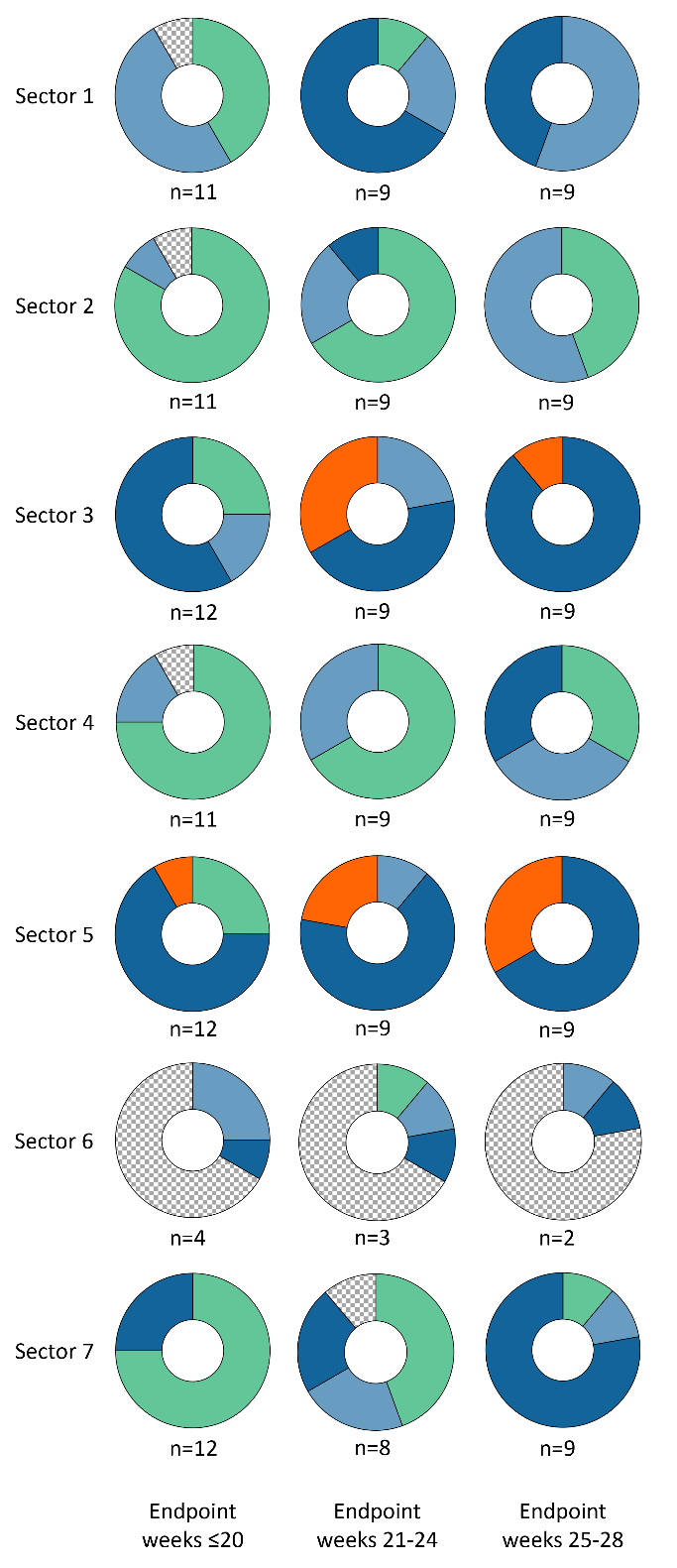

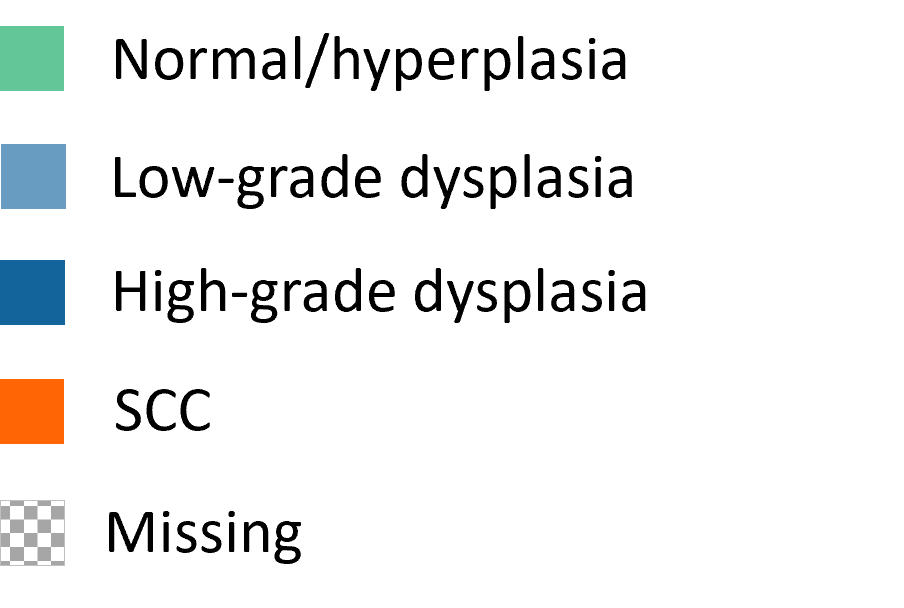


**Supplementary figure S6. Distribution of histopathologic lesions by sector (location on the tongue) and by endpoint in 4NQO-exposed mice.** Tongues were separated into 7 sectors and each sector was assigned a score corresponding to the lesions(s) showing the most severe histopathology. The figure shows the distribution of lesions in the various sectors (1-7) separated by endpoint weeks; ≤20 n=12 (left column), weeks 21-24 n=9 (middle column), and weeks 25-28 n=9 (right column). n represents the total number of sectors that were examined at each endpoint.

**(A)**


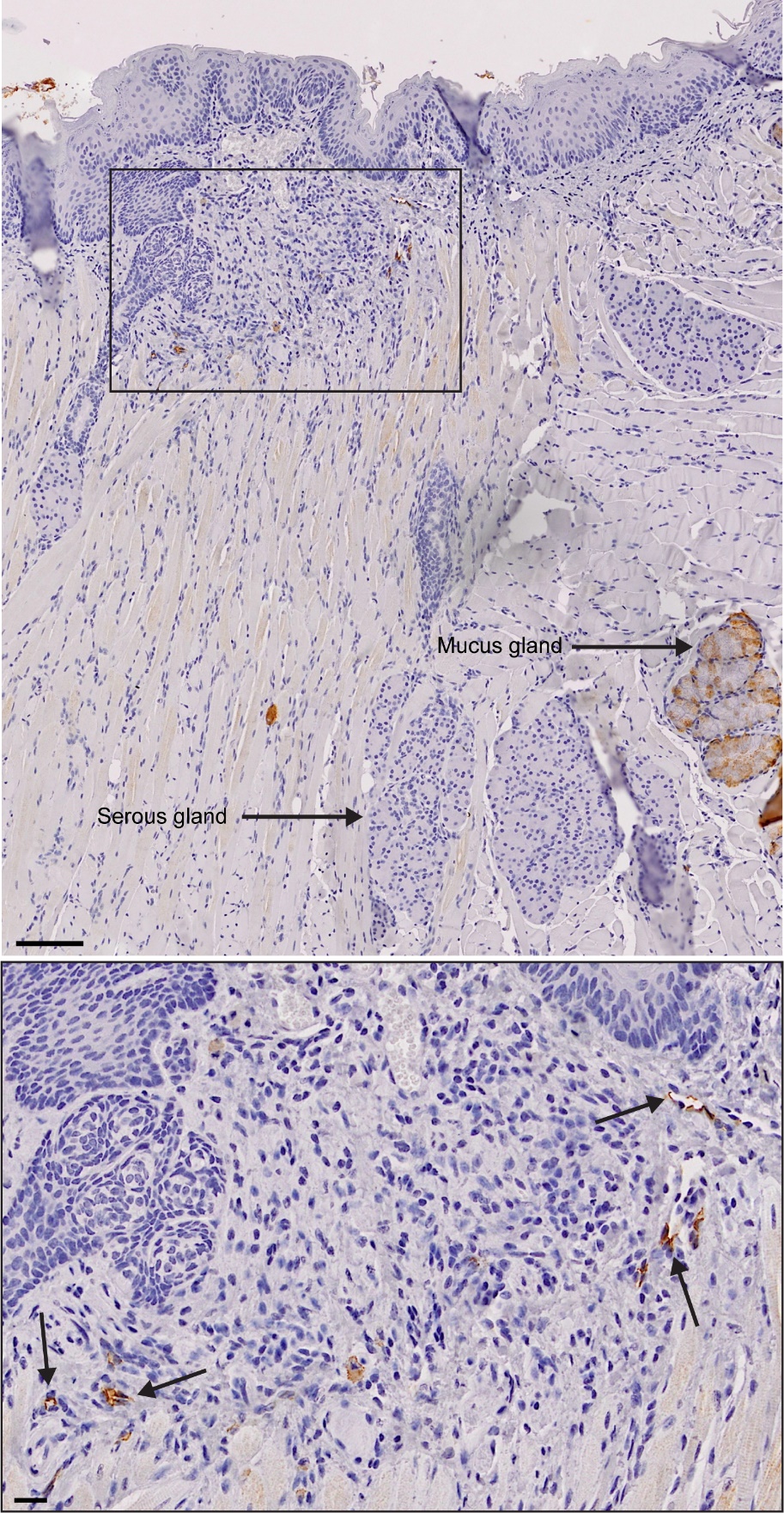


**(B)**

**Supplementary figure S7. PNAd staining in the tongue base (A)** Sagittal section showing the serous- and mucous salivary glands with **(B)** PNAd positive HEVs (arrows) surrounding a structure that is likely an excretory duct of the (serous) glands. Scale bar indicates 100µm (A) or 20µm (B).

**Supplementary figure S8. Immune composition within sectors assigned the same histopathological score.** The figure shows the counts of each marker within sectors scored normal/hyperplasia (n=50), low-grade dysplasia (n=35), high-grade dysplasia (n=67), and SCC (n=8) in 4NQO-exposed mice. Only the sectors where the count of all the markers were available, i.e., not ‘missing’ in any of the consecutively stained sections, were included. Data is log-transformed (log2(y+1)). Error bars indicate mean with SD or median with IQR.

**(B)**

**(A)**


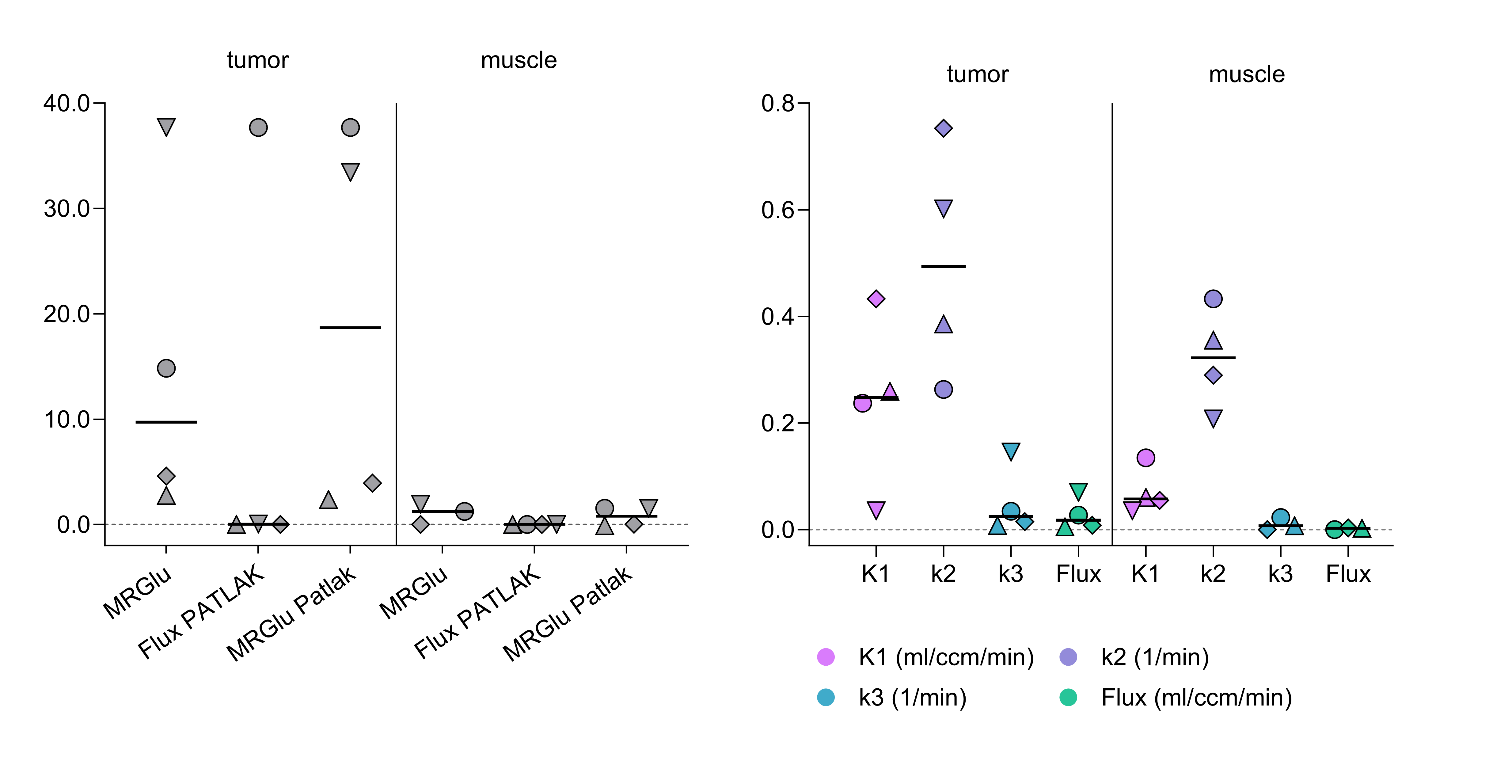


**Supplementary figure S9. PET pharmacokinetic modeling results with [18F]FDG in 4NQO-exposed mice. (A-B)** Rate constants calculated by PET pharmacokinetic modeling using the irreversible 2 tissue compartment model.

## Supplementary tables

**Supplementary table S1**. Distribution of papilloma in tongue sectors 1-7 in 4NQO-exposed mice

|  | **Sector 1** | | | **Sector 2** | | | **Sector 3** | | | **Sector 4** | | | **Sector 5** | | | **Sector 6** | | | **Sector 7** | | |
| --- | --- | --- | --- | --- | --- | --- | --- | --- | --- | --- | --- | --- | --- | --- | --- | --- | --- | --- | --- | --- | --- |
|  | *n=30* | | | *n=30* | | | *n=30* | | | *n=30* | | | *n=30* | | | *n=30* | | | *n=30* | | |
|  | *(No.(%))* | | | *(No.(%))* | | | *(No.(%))* | | | *(No.(%))* | | | *(No.(%))* | | | *(No.(%))* | | | *(No.(%))* | | |
| Papilloma | 2.0 |  | (6.7)***** | 1.0 |  | (3.3) | 2.0 |  | (6.7) | 2.0 |  | (6.7)****** | 6.0 |  | (20.0)******* | 2.0 |  | (6.7) | 5.0 |  | (16.7) |
| No papilloma | 27.0 |  | (90.0) | 28.0 |  | (93.3) | 28.0 |  | (93.3) | 27.0 |  | (90.0) | 24.0 |  | (80.0) | 7.0 |  | (23.3) | 24.0 |  | (80.0) |
| Missing | 1.0 |  | (3.3) | 1.0 |  | (3.3) | 0.0 |  | (0.0) | 1.0 |  | (3.3) | 0.0 |  | (0.0) | 21.0 |  | (70.0) | 1.0 |  | (3.3) |

* two papillomas extended into quadrant 2, ** two papillomas extended into quadrant 6, *** two papillomas extended into quadrant 7.
